# Parliament and the Professions

**Published:** 1921-03-05

**Authors:** 


### Parliament and the Professions.

### Clinical Thermometers.

Sir P. Lloyd-Greame informed Sir H. Nield that the testing of thermometers of all kinds has always been undertaken by the National Physical Laboratory on payment of a fee. The Ministry of Munitions issued an Order in October 1918—for the administration of which the Board of Trade is now responsible—making the testing of clinical thermometers compulsory. This testing, as required by the Order, is performed by the National Physical Laboratory.

### Medical Practitioners and Telephones.

The Postmaster-General, in answer to an inquiry, stated that a preferential tariff for medical practitioners at the expense of other classes of subscribers cannot be justified, but as medical men usually originate comparatively few calls, it is unlikely that the new rates will, apart from exceptional cases, add as much as 100 per cent. to the cost of the service.

### New Remedy for Tuberculosis.

The Minister of Health stated that he had inquired into the Spahlinger cure for consumption, and it is clear that much further investigation and experience will be necessary before accepting the claims made for it. It appears that as long ago as 1913-14 the serum in question was prepared and tried by several hospital physicians, but there is not yet sufficient evidence of its therapeutic value, nor have the trials of it been sufficiently extensive to justify any definite and reliable conclusions. Dr. Addison understood that a report on the method of treatment has been presented to the French Academy of Science, and if and when supplies of the serum become available for investigation in this country he will be prepared at once to facilitate, and co-operate in, an exhaustive examination of its efficacy, both from laboratory and clinical points of view. He understood that a supply of the serum is not likely to be available for this purpose for some months, and that on account of the secrecy maintained as to its constitution and method of preparation it has hitherto been found impracticable for the recognised research laboratories in this country to investigate its properties and effects.

### Navy Surgeons' and Agents' Fees.

Captain Elliott asked the Parliamentary Secretary to the Admiralty when it is intended to revise the scale of fees paid by the Admiralty to surgeons and agents (Article 21, Clause 3), seeing that these fees are below those paid by other Departments, and have not been revised for thirty-five years, notwithstanding the enormous increase in the cost of living.

Sir J. Craig replied that a revised scale of fees is now under consideration, and it is hoped that a decision may be reached and promulgated very shortly.

